# Oat beta-glucan reduces colitis by promoting autophagy flux in intestinal epithelial cells via EPHB6-TFEB axis

**DOI:** 10.3389/fphar.2023.1189229

**Published:** 2023-06-27

**Authors:** Mingyang Xu, Fangmei Ling, Junrong Li, Yidong Chen, Shuang Li, Yiyu Cheng, Liangru Zhu

**Affiliations:** Department of Gastroenterology, Union Hospital, Tongji Medical College, Huazhong University of Science and Technology, Wuhan, China

**Keywords:** colitis, oat beta-glucan, sodium butyrate, EPHB6-TFEB axis, autophagy

## Abstract

Inflammatory bowel disease (IBD) is a group of chronic inflammatory disorders of the gastrointestinal tract, mainly including Crohn’s disease and ulcerative colitis. Epidemiological findings suggest that inadequate dietary fibers intake may be a risk factor for IBD. Oat beta-glucan is a type of fermentable dietary fiber and has been proved to reduce experimental colitis. However, the mechanism remains unclear. The aim of this study was to explore the role and possible mechanism of oat beta-glucan in reducing experimental colitis. We used a dextran sulfate sodium (DSS)-induced mice acute colitis model to explore the potential mechanism of oat beta-glucan in reducing experimental colitis. As a result, oat beta-glucan upregulated the expressions of Erythropoietin-producing hepatocyte receptor B6 (EPHB6) and transcription factor EB (TFEB), promoted autophagy flux and downregulated the expressions of interleukin 1 beta (IL-1β), interleukin 6 (IL-6) and tumor necrosis factor alpha (TNF-α) in intestinal epithelial cells (IECs). The role of the EPHB6-TFEB axis was explored using a lipopolysaccharide-induced HT-29 cells inflammation model. The results revealed that EPHB6 regulated the expression of TFEB, and knockdown of EPHB6 decreased the protein level of TFEB. When EPHB6 or TFEB was knocked down, autophagy flux was inhibited, and the anti-inflammatory effect of sodium butyrate, a main metabolite of oat beta-glucan in the gut, was blocked. In summary, our findings demonstrated that oat beta-glucan reduced DSS-induced acute colitis in mice, promoted autophagy flux via EPHB6-TFEB axis and downregulated the expressions of IL-1β, IL-6 and TNF-α in IECs, and this effect may be mediated by butyrate.

## Introduction

Inflammatory bowel disease (IBD) is a group of chronic inflammatory disorders of the gastrointestinal tract, mainly including Crohn’s disease (CD) and ulcerative colitis (UC) (Torres et al., 2017; Ungaro et al., 2017). The etiology of IBD has not been elucidated and may involve genetic and environmental factors (Ananthakrishnan, 2015). Epidemiological findings suggest that inadequate dietary fibers intake may be one of the potential risk factors for IBD (Levine et al., 2018).

Chronic deficiency of dietary fibers may decrease intestinal microbial diversity and reduce short-chain fatty acids (SCFAs) production, disrupting the intestinal epithelial barrier and promoting the occurrence and development of IBD (Martini et al., 2017; Makki et al., 2018). SCFAs are key metabolites of dietary fibers in the gut and mainly include acetate, propionate and butyrate. Acetate and propionate are mainly metabolized in the periphery, while butyrate is mainly metabolized in the colonic epithelial cells and is an important source of energy for them (Koh et al., 2016; Boets et al., 2017).

Studies have suggested that butyrate not only provides energy to intestinal epithelial cells (IECs), but also regulates their function and proliferation (Hamer et al., 2008; Couto et al., 2020; Hodgkinson et al., 2022). Normally, IECs maintain intestinal homeostasis by forming physical and biochemical barriers, and their functional defects and abnormal death could disrupt the integrity of the intestinal epithelial barrier and increase intestinal permeability (Peterson and Artis, 2014; Patankar and Becker, 2020).

Dietary fibers come from a wide range of sources, and not all types could reduce intestinal inflammation (Wong et al., 2016). Oat beta-glucan is a fermentable dietary fiber that can be metabolized by intestinal microbes to produce SCFAs (Gill et al., 2021). There is evidence that alterations in the composition of intestinal microbes in patients with CD and UC are mainly characterized by reduced abundance of butyrate-producing bacteria such as *Faecalibacterium prausnitzii* (Joossens et al., 2011; Machiels et al., 2014). Supplying dietary fibers that promotes butyrate production is a potential strategy to prevent or treat colitis. Oat beta-glucan has been confirmed to increase butyrate level in the gut and reduce experimental colitis in mice, but the exact mechanism remains unclear (Liu et al., 2015; Bai et al., 2021).

Erythropoietin-producing hepatocyte receptor B6 (EPHB6) is a receptor tyrosine kinase that regulates the intestinal epithelial barrier, and knockdown of EPHB6 significantly increases intestinal permeability in mice (Li et al., 2020). Moreover, EPHB6 has been reported to regulate the activation of transcription factor EB (TFEB) (Zangrossi et al., 2021). TFEB is an important regulatory transcription factor of autophagy and is involved in regulating not only lysosomal biogenesis but also the expressions of other autophagy-related genes (Sardiello et al., 2009; Settembre and Ballabio, 2011; Settembre et al., 2011). It has been reported that TFEB may regulate the inflammatory response by regulating the autophagy-lysosome pathway and that specific knockout of TFEB in IECs aggravates dextran sulfate sodium (DSS)-induced colitis (Murano et al., 2017; Brady et al., 2018).

**FIGURE 1:**
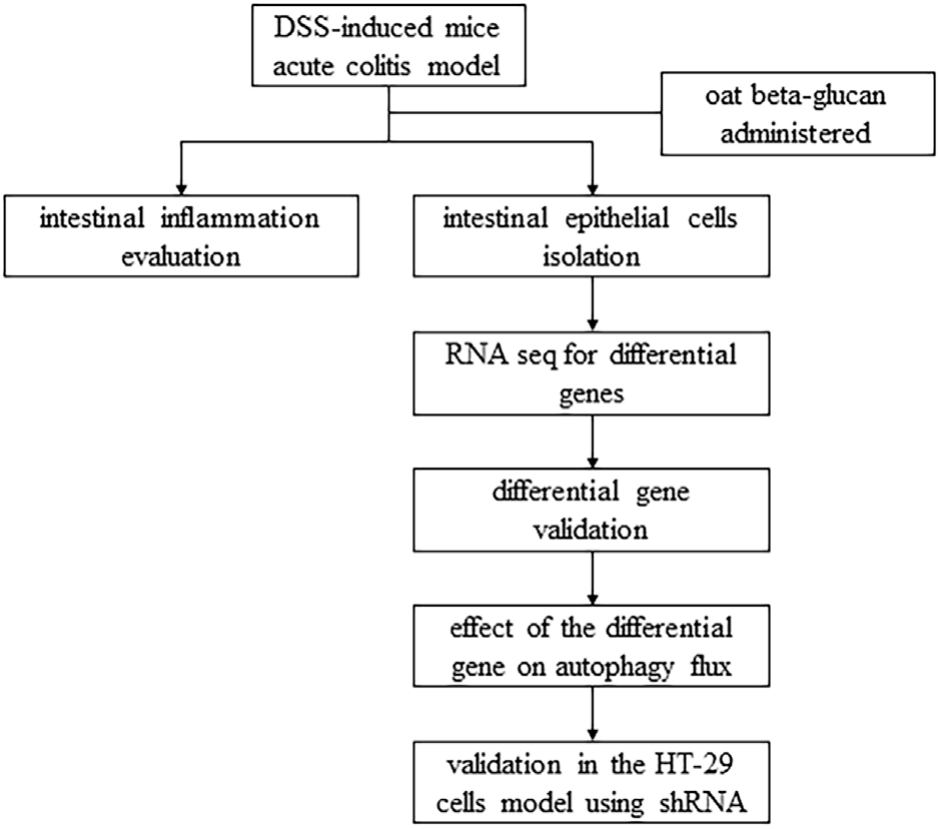
Flow chart of the study.

In this study, we used a DSS-induced mice acute colitis model to explore the potential mechanism of oat beta-glucan in reducing experimental colitis and a lipopolysaccharide-induced HT-29 cells inflammation model to explore the role of the EPHB6-TFEB axis in the mechanism of oat beta-glucan in reducing experimental colitis, hoping to provide more evidence to support the prevention or treatment of IBD with oat beta-glucan (Figure 1).

## Materials and methods

### Animals

C57BL/6 specific pathogen-free male mice (8-week-old, weighting 21–23 g) were purchased from Beijing Vital River Laboratory Animal Technology Co., Ltd., (Beijing, China). Mice were housed in the barrier system experimental area of the Experimental Animal Center of Huazhong University of Science and Technology (Wuhan, China). The experimental area was at constant temperature and humidity, with fixed light hours, and experimental animals had free access to adequate food and water. 60 mice were randomly divided into 4 groups of 15 mice each: control group (Control), oat beta-glucan group (βG), colitis group (DSS) and oat beta-glucan treatment group (DSS+βG). The modeling period was 2 weeks. In the first week, the Control and DSS groups were given AIN-93M purified diet (Xietong, Nanjing, China), and the βG and DSS+βG groups were given AIN-93M purified diet with 10% (w/w) oat beta-glucan (purity: 80%, replace corn starch, Xietong, Nanjing, China). In the second week, the Control group was given AIN-93M purified diet, the βG group was given AIN-93M purified diet with 10% (w/w) oat beta-glucan, the DSS group was given AIN-93M purified diet and induced acute colitis by administration of 3% (w/v) DSS (MP Biomedicals, IIIkirch, France) in drinking water for 7 days, and the DSS+βG group was given AIN-93M purified diet with 10% (w/w) oat beta-glucan and induced acute colitis by administration of 3% (w/v) DSS in drinking water for 7 days. At the end of modeling, mice were sacrificed, and the colon of about 0.5 mm near the anal side was fixed in 4% paraformaldehyde (Pinuofei, Wuhan, China) for 24 h and embedded in paraffin by Wuhan Pinuofei Biological Technology Co., Ltd., (Wuhan, China), and the rest of the colon tissues were used to isolate intestinal epithelial cells (IECs) immediately for subsequent study.

### Disease activity index scoring

Disease activity index (DAI) score consists of three components: body weight, stool consistency and stool blood, which were recorded daily during the modeling period. The standard of weight loss score: score 0 for no weight loss, score 1 for weight loss within 1%–5%, score 2 for weight loss within 5%–10%, score 3 for weight loss within 10%–15% and score 4 for weight loss more than 15%. The standard of stool consistency score: score 0 for normal, score 2 for loose stool and score 4 for diarrhea. The standard of stool blood score: score 0 for negative occult blood, score 2 for positive occult blood and score 4 for rectal bleeding.

### Cell culture and plasmid transfection

HT-29 cells, a human colorectal adenocarcinoma cell line with epithelial morphology, were obtained from the laboratory of Department of Gastroenterology, Union Hospital, Tongji Medical College, Huazhong University of Science and Technology (Wuhan, China) and cultured in RPMI 1640 medium (Hycezmbio, Wuhan, China) supplemented with 10% (v/v) fetal bovine serum (Gbico, United States) and 1% (v/v) penicillin-streptomycin (Beyotime, Shanghai, China) in the incubator at 37°C and 5% CO_2_ concentration. Knock down EPHB6 and TFEB in HT-29 cells using *EPHB6* specific short hairpin RNA (shRNA) plasmid (Genechem, Shanghai, China) and *TFEB* specific shRNA plasmid (Genechem, Shanghai, China), respectively. Cells were seeded in 24-well plates 1 day before transfection, and the complete medium was replaced 2 h before transfection. Cells were then transfected with the transfection complex of 0.5 μg plasmid carrying shRNA, 50 μL Opti-MEM (Gbico, United States) and 0.5 μL transfection reagent (Neofect, Beijing, China) in 500 μL complete medium and cultured for 48 h. Inflammation was induced using lipopolysaccharide (LPS). There were 6 groups: control group (Control), LPS group (LPS), sodium butyrate (NaB) treatment group (LPS + NaB), empty plasmid vector group (shCtrl), EPHB6 knockdown group (shEPHB6) and TFEB knockdown group (shTFEB). The LPS group was given LPS (10 μg/mL, Solarbio, Beijing, China), the LPS + NaB group was given LPS (10 μg/mL) and NaB (0.5 mmol/L, Beyotime, Shanghai, China), and the shCtrl, shEPHB6 and shTFEB groups were given LPS (10 μg/mL) and NaB (0.5 mmol/L). Intervention duration for all groups was 24 h.

### Hematoxylin-eosin staining

Hematoxylin-eosin staining (H&E) was performed by Wuhan Pinuofei Biological Technology Co., Ltd., (Wuhan, China). Images were captured by the optical microscope (Olympus, Japan).

### Immunohistochemistry

Immunohistochemistry (IHC) assay for interleukin 1 beta (IL-1β), interleukin 6 (IL-6) and tumor necrosis factor alpha (TNF-α) was performed by Wuhan Pinuofei Biological Technology Co., Ltd., (Wuhan, China). Images were captured by the optical microscope (Olympus, Japan).

### Immunofluorescence

Sections of colon tissues were deparaffinized and rehydrated. Antigens were retrieved by treatment of slides with citrate buffer (10 mmol/L, pH 6.0, Servicebio, Wuhan, China) for 2 min at 100°C, and then slides were washed in PBS for 3 times, 5 min each time. Non-specific staining was blocked by incubation of slides in normal donkey serum (Antgene, Wuhan, China) for 30 min at room temperature. Slides were then incubated with anti-microtubule associated protein 1 light chain 3 beta (LC3B) primary antibody (1:200, ABclonal, Wuhan, China) overnight at 4°C. After washed in PBS for 3 times, 5 min each time, slides were incubated with Alexa Fluor 488-conjugated secondary antibody (1:200, Servicebio, Wuhan, China) for 1 h at room temperature. Slides were then washed in PBS for 3 times, 5 min each time, and finally covered with anti-fade mounting medium with DAPI (Antgene, Wuhan, China). Images were captured by the fluorescence microscope (Olympus, Japan).

### IECs isolation

The colon was washed in cold phosphate buffered saline (PBS) (Gbico, United States) and then cut into 5 mm tissue segments. Tissues were incubated in 15 mL mixture of ethylene diamine tetraacetic acid (EDTA) (Sigma, Germany) and dithiothreitol (Roche, Switzerland) and shaken slowly for 75 min at 4°C. The concentration of EDTA in the mixture was 8 mmol/L, and the concentration of dithiothreitol was 1 mmol/L. Tissues were then removed and washed in cold PBS and shaken vigorously for several times. The tissue suspension was filtered quickly through a 70 μm filter and centrifuged at 800 g for 5 min at 4°C. The sediment was enriched with IECs.

### RNA sequencing

5 samples of IECs were randomly selected from the DSS+βG and DSS groups by random lottery method and sent for RNA sequencing. RNA quantification and qualification, cDNA libraries preparation, clustering and sequencing and data analyses were performed by Metware Biotechnology Co., Ltd., (Wuhan, China). The cDNA libraries were sequenced on the Illumina sequencing platform.

### RNA isolation and quantitative real-time polymerase chain reaction

Total RNA was isolated from IECs or HT-29 cells using RNA-easy lsolation Reagent (Vazyme, Nanjing, China). 500 ng of total RNA was then reversely transcribed to cDNA using HiScript II qRT SuperMix II (Vazyme, Nanjing, China). Quantitative real-time polymerase chain reaction (qRT-PCR) was performed by LightCycler 480 qRT-PCR instrument (Roche, Switzerland) using AceQ qPCR SYBR Green Master Mix (Vazyme, Nanjing, China). Primers were synthesized by Tsingke Biotechnology Co., Ltd., (Beijing, China). The relative expressions were calculated by the 2^−ΔΔCt^ method using *Actb* or *ACTB* as an internal control. Primer sequences were shown in Table 1.

**TABLE 1:** Primer sequences.

Gene name	Primer sequences (5′-3′)
*Il1β* (mouse)	Forward: GAA​ATG​CCA​CCT​TTT​GAC​AGT​G
	Reverse: TGG​ATG​CTC​TCA​TCA​GGA​CAG
*Il6* (mouse)	Forward: TAG​TCC​TTC​CTA​CCC​CAA​TTT​CC
	Reverse: TTG​GTC​CTT​AGC​CAC​TCC​TTC
*Tnfα* (mouse)	Forward: CCT​GTA​GCC​CAC​GTC​GTA​G
	Reverse: GGG​AGT​AGA​CAA​GGT​ACA​ACC​C
*Actb* (mouse)	Forward: GTG​ACG​TTG​ACA​TCC​GTA​AAG​A
	Reverse: GCC​GGA​CTC​ATC​GTA​CTC​C
*IL1β* (human)	Forward: ATG​ATG​GCT​TAT​TAC​AGT​GGC​AA
	Reverse: GTC​GGA​GAT​TCG​TAG​CTG​GA
*IL6* (human)	Forward: ACT​CAC​CTC​TTC​AGA​ACG​AAT​TG
	Reverse: CCA​TCT​TTG​GAA​GGT​TCA​GGT​TG
*TNFα* (human)	Forward: GAG​GCC​AAG​CCC​TGG​TAT​G
	Reverse: CGG​GCC​GAT​TGA​TCT​CAG​C
*ACTB* (human)	Forward: CAT​GTA​CGT​TGC​TAT​CCA​GGC
	Reverse: CTC​CTT​AAT​GTC​ACG​CAC​GAT

### Western blot

Total protein was extracted from IECs or HT-29 cells using cold RIPA buffer (Servicebio, Wuhan, China) with 1% (v/v) PMSF (Solarbio, Beijing, China). The concentration of the total protein was measured using the BCA protein assay kit (Aspen, Wuhan, China). Protein samples were then mixed with SDS-PAGE sample loading buffer (5X, Beyotime, Shanghai, China) and boiled for 10 min at 100°C. The total protein (15 μg/well) was separated by 12.5% SDS-PAGE gel (Epizyme, Shanghai, China) electrophoresis and then transferred to PVDF membranes (Millipore, Germany). The membranes were blocked with protein free rapid blocking buffer (Epizyme, Shanghai, China) for 10 min at room temperature and then incubated with anti-EPHB6 primary antibody (1:1000, ABclonal, Wuhan, China), anti-TFEB primary antibody (1:1000, ABclonal, Wuhan, China), anti-LC3B primary antibody (1:1000, ABclonal, Wuhan, China), anti-p62 primary antibody (1:1000, ABclonal, Wuhan, China) and anti-ACTB primary antibody (1:1000, Servicebio, Wuhan, China), respectively, overnight at 4°C. After washed in tris buffered saline with 0.1% (v/v) tween-20 (TBST) (Servicebio, Wuhan, China) for 3 times, 10 min each time, the membranes were incubated with HRP-conjugated secondary antibody (1:3000, Antgene, Wuhan, China) for 1 h at room temperature. The membranes were then washed in TBST for 3 times, 10 min each time. Proteins were finally visualized by chemiluminescence imaging system (Clinx, Shanghai, China) using ECL (Vazyme, Nanjing, China). The expressions of the proteins were analyzed using ImageJ software.

### Statistical analyses

Shapiro-Wilk test was used to check the normal distribution. One-way ANOVA was used to analyze the differences between multiple groups. Statistical analyses and figures were performed using GraphPad Prism 9 (GraphPad Software, San Diego, California). Data analyses and figure creation for RNA sequencing were performed using the Metware Cloud (Wuhan, China), a free online platform for data analysis (https://cloud.metware.cn). *p*-values < 0.05 were considered statistically significant, and all *p*-values were two-tailed.

## Results

### Oat beta-glucan reduced DSS-induced acute colitis in mice

**FIGURE 2:**
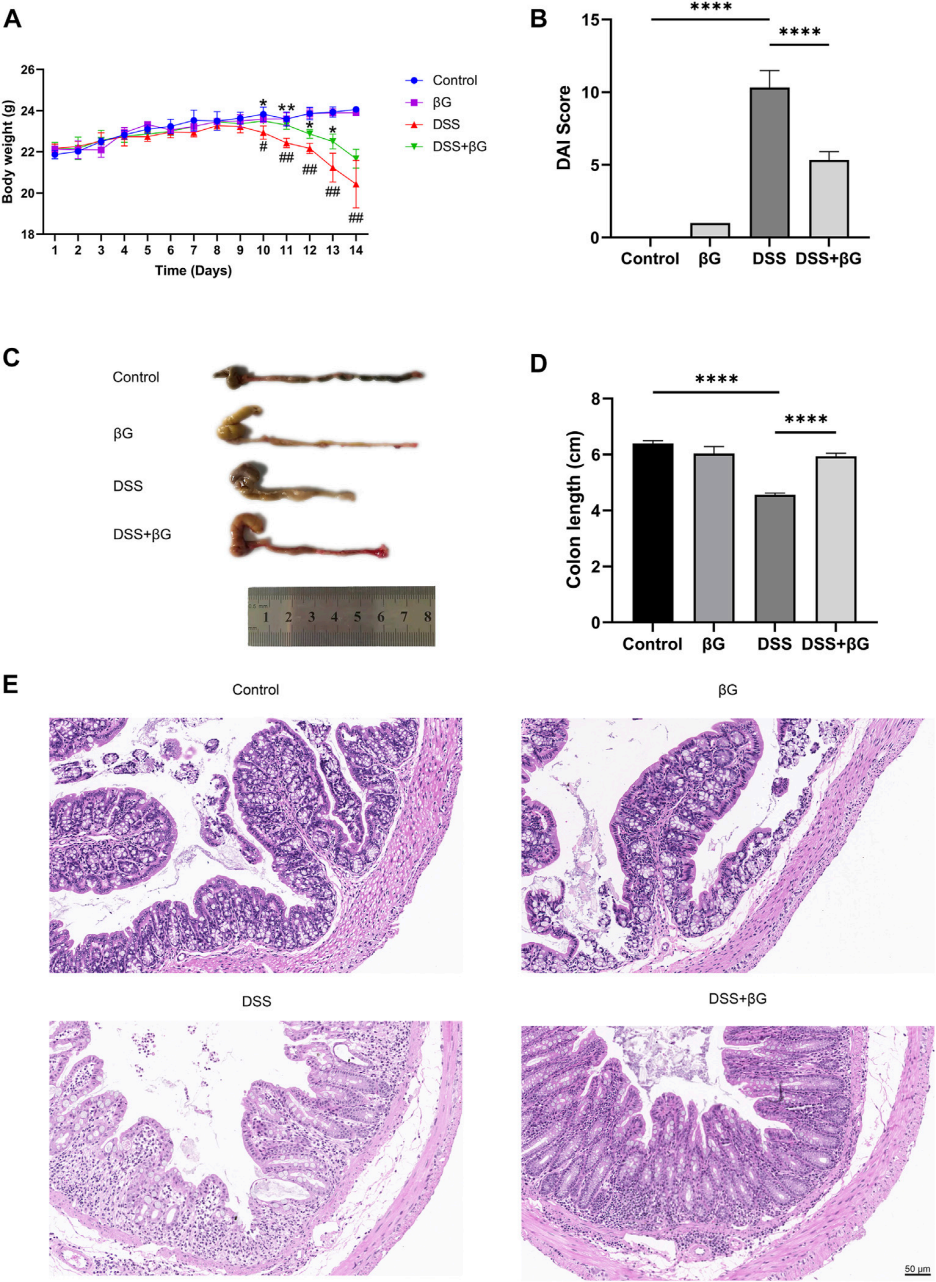
Oat beta-glucan reduced DSS-induced acute colitis in mice. **(A)** Body weight. **(B)** DAI score (*n* = 3). **(C,D)** Colon length (*n* = 3). **(E)** H&E of the colon tissues. (# DSS vs. Control, * DSS+βG vs. DSS, #*p* < 0.05, ##*p* < 0.01, *p < 0.05, **p < 0.01; ****p < 0.0001).

Oat beta-glucan reversed weight loss, the increase of DAI scores and colon shortening in mice with DSS-induced acute colitis (Figures 2A–D). H&E showed less destruction of colon glands in the DSS+βG group than that in the DSS group (Figure 2E).

According to the results, the administration of oat beta-glucan did not cause significant effects on body weight and the length and histopathology of the colon in mice. Therefore, the βG group was not considered in the subsequent study.

**FIGURE 3:**
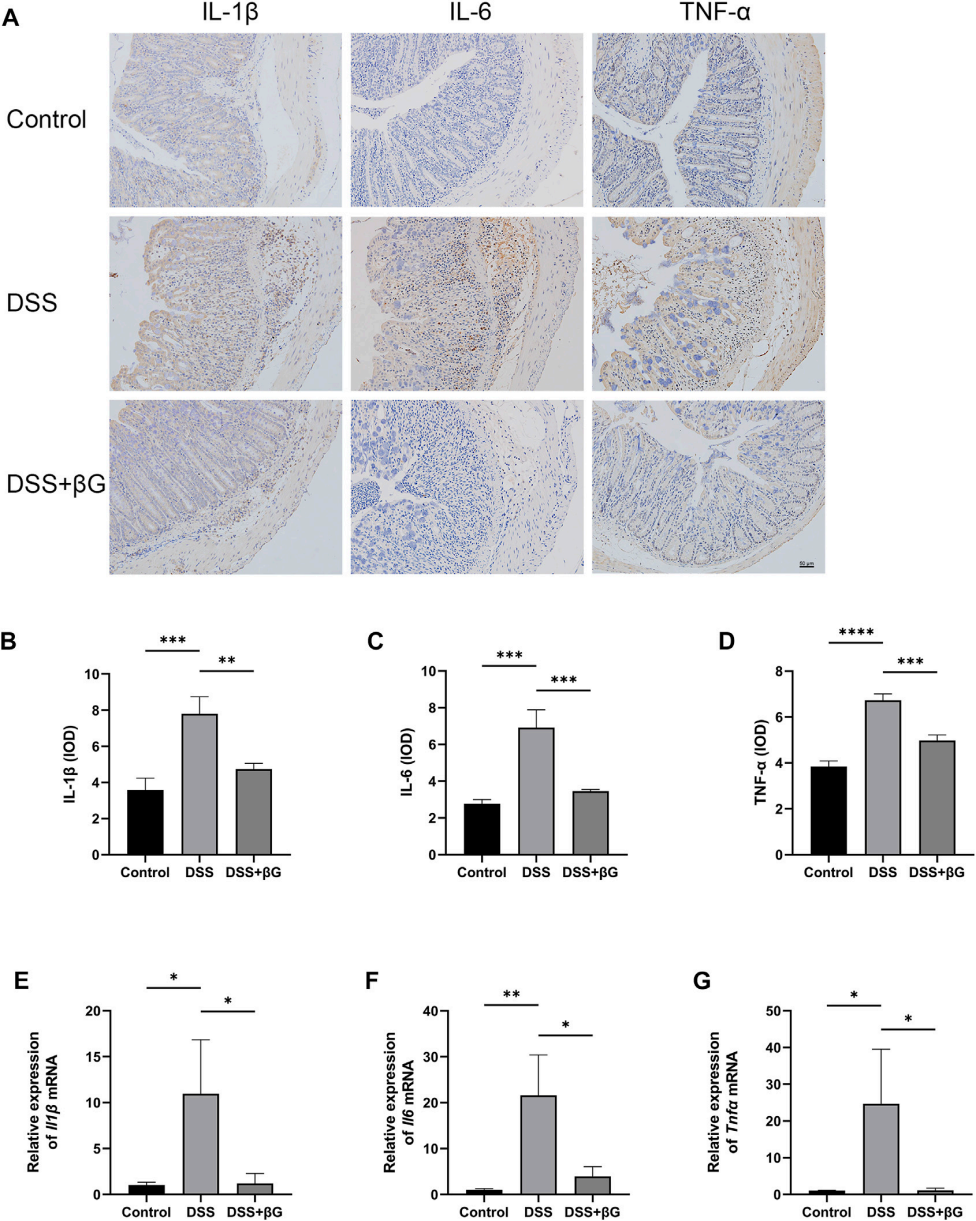
Oat beta-glucan downregulated the expressions of IL-1β, IL-6 and TNF-α in intestinal epithelial cells of mice with acute colitis. **(A–D)** Detection of IL-1β, IL-6 and TNF-α expressions in the colon tissues by IHC (*n* = 3). **(E–G)** Detection of *Il1β*, *Il6* and *Tnfα* mRNA expressions in intestinal epithelial cells by qRT-PCR (*n* = 3). (*p < 0.05, **p < 0.01, ***p < 0.001, ****p < 0.0001).

IHC result showed that the expressions of IL-1β, IL-6 and TNF-α in the colon tissues were significantly lower in the DSS+βG group than those in the DSS group (Figures 3A–D). qRT-PCR result further confirmed that oat beta-glucan significantly downregulated the expressions of *Il1β*, *Il6* and *Tnfα* mRNA in IECs (Figures 3E–G).

### Oat beta-glucan upregulated EPHB6 expression in IECs

**FIGURE 4:**
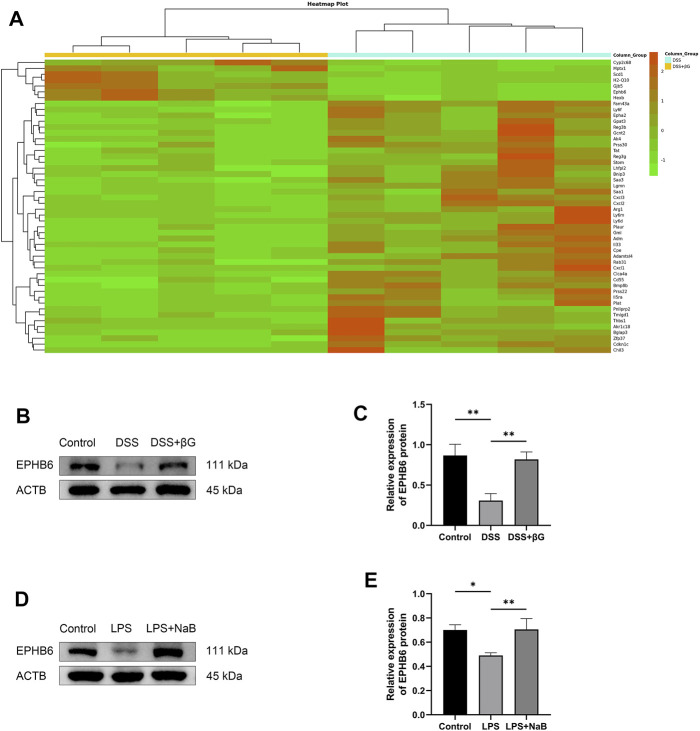
Oat beta-glucan and NaB upregulated the expressions of EPHB6. **(A)** Heat map of differential genes (top 50) in intestinal epithelial cells between the DSS+βG and DSS groups (*n* = 5). **(B,C)** Detection of EPHB6 expression in intestinal epithelial cells by WB (*n* = 3). **(D,E)** Detection of EPHB6 expression in HT-29 cells by WB (*n* = 3). (*p < 0.05, **p < 0.01).

RNA sequencing was used to analyze the differences in gene expression of IECs between the DSS+βG and DSS groups, and a total of 185 genes were revealed to be significantly different, of which the top 50 were shown in Figure 4A. The result showed that oat beta-glucan significantly upregulated the expression of *Ephb6* in IECs of mice with acute colitis. Western blot (WB) result showed that the expression of EPHB6 in IECs was significantly higher in the DSS+βG group than that in the DSS group, which was consistent with the RNA sequencing result (Figures 4B, C).

NaB was administered to the LPS-induced HT-29 cells inflammation model to assess the effect of butyrate on EPHB6 expression, and the result showed that NaB also significantly upregulated EPHB6 expression (Figures 4D, E).

### Oat beta-glucan upregulated TFEB expression and promoted autophagy flux in IECs

**FIGURE 5:**
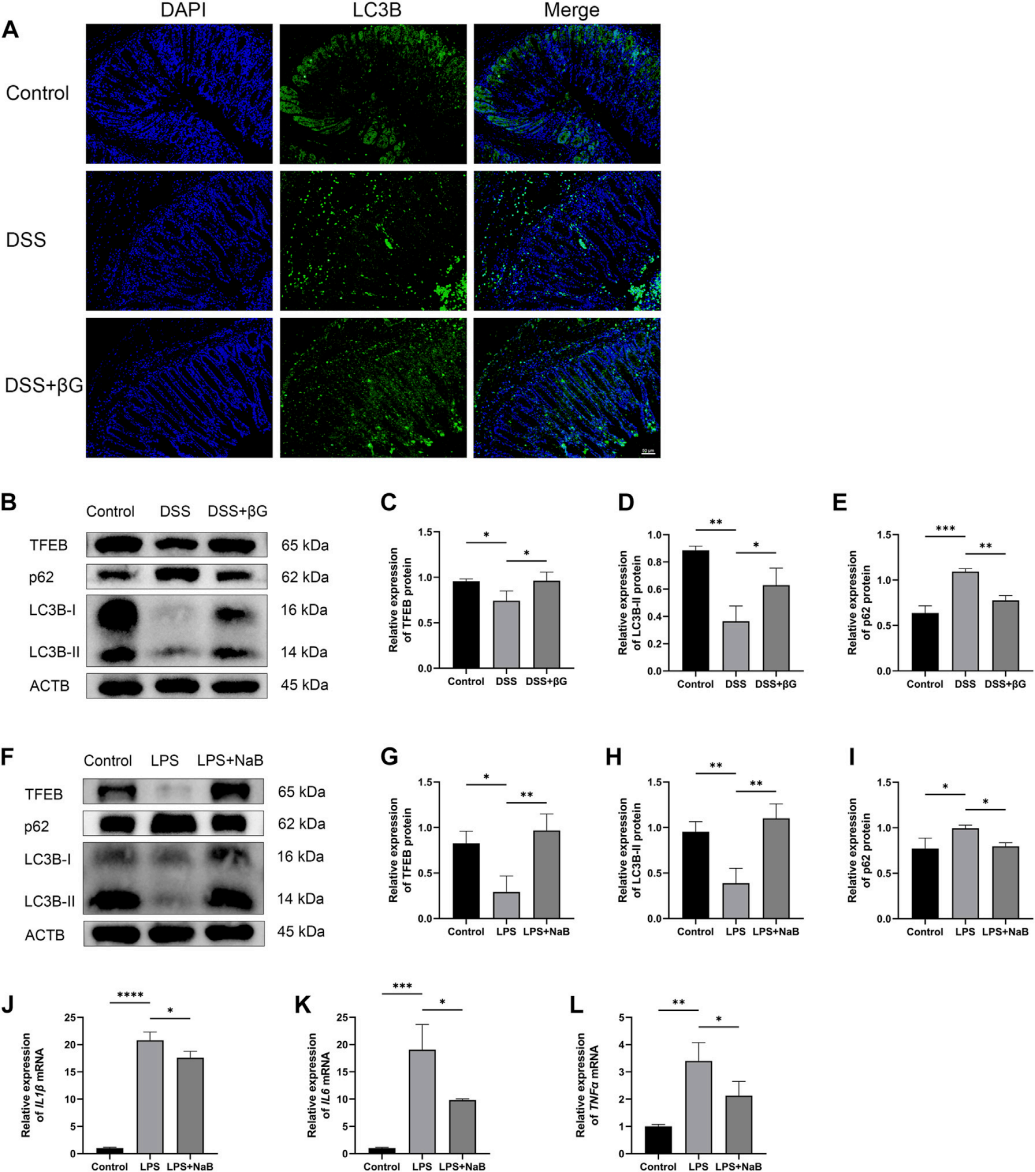
Oat beta-glucan and NaB promoted autophagy flux. **(A)** Detection of LC3B expression in the colon tissues by IHC. **(B–E)** Detection of TFEB, LC3B and p62 expressions in intestinal epithelial cells by WB (*n* = 3). **(F–I)** Detection of TFEB, LC3B and p62 expressions in HT-29 cells by WB (*n* = 3). **(J–L)** Detection of *IL1β*, *IL6*, and *TNFα* mRNA expressions in HT-29 cells by qRT-PCR (*n* = 3). (*p < 0.05, **p < 0.01, ***p < 0.001, ****p < 0.0001).

LC3B was detected in the colon tissues by immunofluorescence for preliminary assessment of the autophagy level. The result showed that the expression of LC3B in the colon epithelium was higher in the DSS+βG group than that in the DSS group (Figure 5A).

WB result showed that the expressions of TFEB and LC3B in IECs were significantly higher in the DSS+βG group than that in the DSS group, and the expression of p62 was significantly lower in the DSS+βG group than that in the DSS group (Figures 5B–E).

The results of *in vitro* experiments showed that NaB significantly upregulated the expressions of TFEB and LC3B, decreased the protein level of p62 (Figures 5F–I) and downregulated the relative expressions of *IL1β*, *IL6* and *TNFα* mRNA (Figures 5J–L).

### EPHB6-TFEB axis mediated the promotion of autophagy flux in IECs and the anti-inflammatory effect of NaB

**FIGURE 6:**
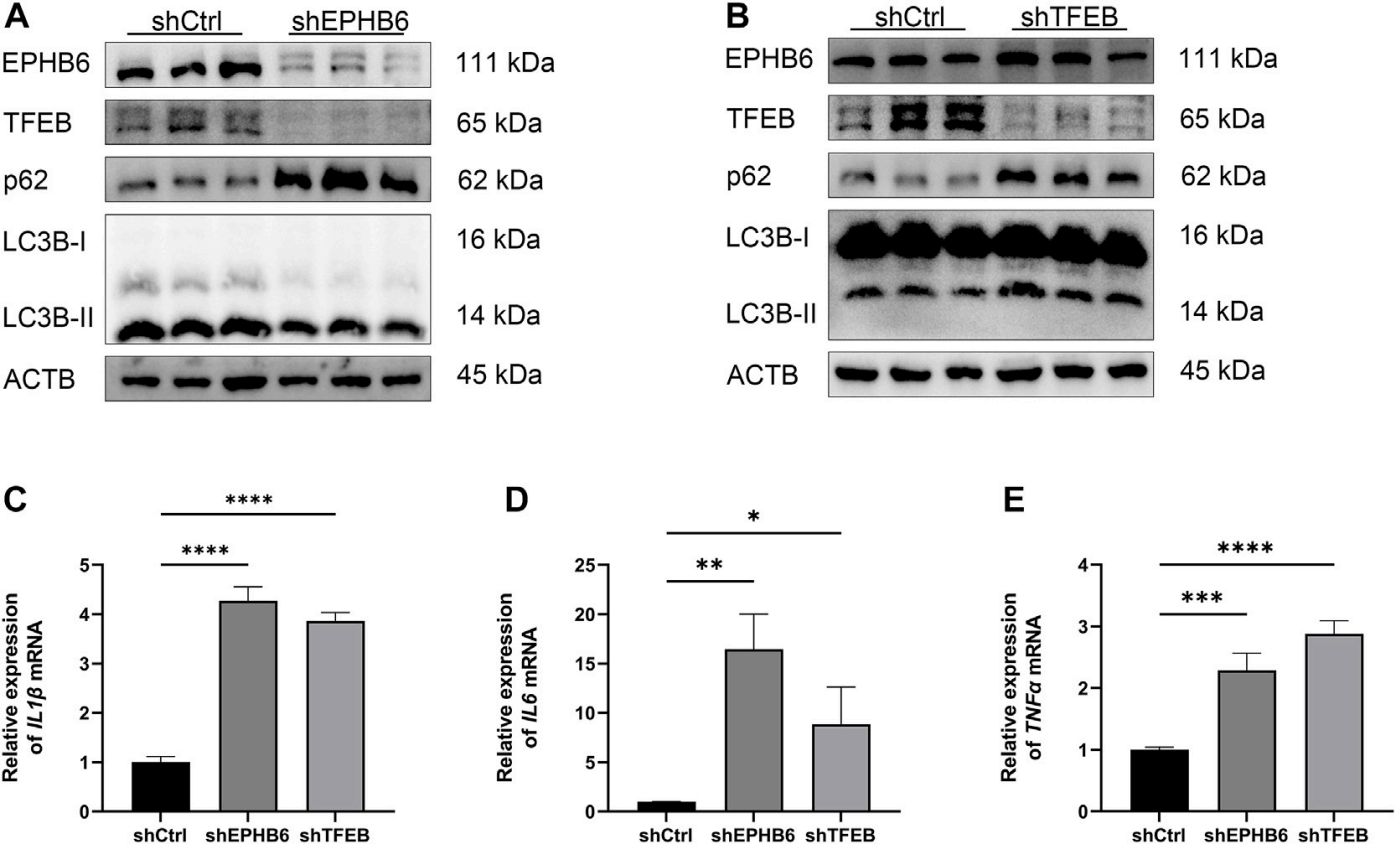
EPHB6-TFEB axis mediated the promotion of autophagy flux and the anti-inflammatory effect of NaB. **(A)** Detection of EPHB6, TFEB, LC3B-II and p62 expressions in the shCtrl and shEPHB6 groups by WB (*n* = 3). **(B)** Detection of EPHB6, TFEB, LC3B-II and p62 expressions in the shCtrl and shTFEB groups by WB (*n* = 3). **(C–E)** Detection of *IL1β*, *IL6* and *TNFα* mRNA expressions in HT-29 cells by qRT-PCR (*n* = 3). (*p < 0.05, **p < 0.01, ***p < 0.001, ****p < 0.0001).

WB result showed that the expression of TFEB was significantly lower in the shEPHB6 group compared with the shCtrl group, while the expression of LC3B-II was not significantly different and the expression of p62 was significantly higher in the shEPHB6 group (Figure 6A). Between the shTFEB and shCtrl groups, there was no significant difference in the expressions of EPHB6 and LC3B-II, while the expression of p62 was significantly higher in the shTFEB group than that in the shCtrl group (Figure 6B).

qRT-PCR result showed that the relative expressions of *IL1β*, *IL6* and *TNFα* mRNA were significantly higher in the shEPHB6 and shTFEB groups than those in the shCtrl group (Figures 6C–E).

## Discussion

Oat beta-glucan, a type of fermentable dietary fiber, has been shown to reduce experimental colitis, and this effect is associated with its increased butyrate level in the gut (Liu et al., 2015; Bai et al., 2021). Our results were consistent with previous studies, in which oat beta-glucan given in the diet reduced DSS-induced acute colitis in mice. It reversed weight loss, the increase of DAI scores and colon shortening in mice with acute colitis, attenuated the destruction of colon glands and immune cells infiltration and downregulated the expressions of IL-1β, IL-6 and TNF-α in colon tissues.

IBD is characterized by impaired intestinal epithelial barrier, which could increase intestinal permeability (Camilleri, 2019). EPHB6 belongs to the EPH family of receptor tyrosine kinases. EPH receptors play an important role in regulating cell proliferation, differentiation and migration, and there is evidence that EPH receptors regulate the intestinal epithelial barrier, maintain intestinal homeostasis and are involved in the occurrence and development of several chronic inflammatory diseases (Batlle et al., 2002; Pasquale, 2005; Holmberg et al., 2006; Pasquale, 2008; Coulthard et al., 2012; Kania and Klein, 2016). EPHB6 has no kinase activity but acts as a molecular switch to regulate the signaling of other EPH receptors (Liang et al., 2019; Strozen et al., 2021). We used RNA sequencing to reveal that oat beta-glucan significantly upregulated *Ephb6* expression in IECs of mice with acute colitis. And our findings demonstrated that oat beta-glucan upregulated TFEB expression as well, which was regulated by EPHB6.

TFEB is known to regulate autophagy. Autophagy dysfunction may lead to a variety of diseases including IBD (Larabi et al., 2020; Klionsky et al., 2021). Genome-wide association studies show that polymorphisms in several autophagy-related genes, such as *ATG16L1*, are associated with susceptibility to IBD (Hampe et al., 2007; Parkes et al., 2007; Rioux et al., 2007). Previous studies have reported that TFEB regulates autophagy by driving the expressions of lysosomal and autophagy-related gene, and autophagy plays an important role in regulating the intestinal epithelial barrier and maintaining intestinal homeostasis by reducing proinflammatory cytokines production, removing intracellular pathogenic microbes and protecting IECs from cellular stress-induced injury (Patel and Stappenbeck, 2013; Baxt and Xavier, 2015; Lassen and Xavier, 2018; Wu et al., 2019; Foerster et al., 2022). So, we speculated whether TFEB might also be involved in driving the expressions of genes such as antimicrobial defense or tissue repair in IECs, but more studies would be required to confirm this. Upregulation of TFEB expression would contribute to promote autophagy flux, which was proved in our study. Administration of oat beta-glucan promoted autophagy flux in IECs of mice with acute colitis while upregulating TFEB. Promotion of autophagy flux in IECs could reduce TNF-α-induced cell death, thereby maintaining the intestinal epithelial barrier and reducing intestinal permeability (Pott et al., 2018; Saha et al., 2022).

Our results showed that DSS-induced acute colitis led to impaired autophagy flux in IECs, and that administration of oat beta-glucan could promote autophagy flux in IECs, downregulate the expressions of IL-1β, IL-6 and TNF-α and reduce intestinal inflammation. We observed in the RNA sequencing results as well that genes related to acute inflammatory response, leukocyte migration, leukocyte chemotaxis and TNF signaling pathway were downregulated in IECs of the DSS+βG group compared to the DSS group. This further supported that oat beta-glucan alleviated DSS-induced inflammation in the gut. Like the effect of oat beta-glucan, NaB promoted autophagy flux and suppressed LPS-induced inflammation *in vitro*. When EPHB6 or TFEB was knocked down in the LPS-induced HT-29 cells inflammation model, promotion of autophagy flux by NaB was then inhibited, and levels of proinflammatory cytokines increased. This suggested that EPHB6-TFEB axis played an important role in promotion of autophagy flux and downregulation of proinflammatory cytokines by oat beta-glucan. Moreover, this study had some limitations. Although previous studies have confirmed that oat beta-glucan could significantly increase butyrate level in the feces of mice (Liu et al., 2015), in our study, we did not collect fecal samples from mice and did not detect the level of butyrate and inflammatory markers in the stool or anti-inflammatory markers in the gut.

In summary, we demonstrated that oat beta-glucan could promote autophagy flux in IECs via EPHB6-TFEB axis and downregulate the expressions of proinflammatory cytokines, thereby reducing DSS-induced acute colitis in mice, and this effect may be mediated by butyrate.

## Data Availability

The datasets presented in this study can be found in online repositories. The names of the repository/repositories and accession number(s) can be found below: https://www.ncbi.nlm.nih.gov/, PRJNA924951.
